# Fulminant cerebral fat embolism syndrome initially sparing the lungs after long-bone fractures: a case report of refractory status epilepticus

**DOI:** 10.3389/fmed.2026.1893003

**Published:** 2026-07-01

**Authors:** Jian Lan, Xingdong Li, Xuesheng Jian, Qiang Zhong, Xijiang Zhang, Cheng Zheng

**Affiliations:** 1 Department of Critical Care Medicine, Taizhou Municipal Hospital (Affiliated Taizhou University Hospital), School of Medicine, Taizhou University, Taizhou, Zhejiang, China; 2 Department of Emergency Medicine, Mangya People's Hospital, Mangya, Qinghai, China; 3 Department of Emergency Medicine, Taizhou Municipal Hospital (Affiliated Taizhou University Hospital), School of Medicine, Taizhou University, Taizhou, Zhejiang, China; 4 Taizhou Key Laboratory of Infection and Tumor Immunology, Taizhou Municipal Hospital, Taizhou, China

**Keywords:** cerebral fat embolism syndrome, normal early oxygenation, poor neurological outcome, pulmonary-sparing, starfield pattern

## Abstract

**Background:**

Cerebral fat embolism syndrome (CFES) usually occurs within fat embolism syndrome, classically characterized by hypoxemia, neurological abnormalities, and petechial rash. Pulmonary-sparing CFES is uncommon and may delay diagnosis.

**Case presentation:**

A 23-year-old man sustained multiple lower-extremity long-bone fractures after a traffic accident. After emergency orthopedic surgery, he was fully conscious with preserved oxygenation. Approximately 12 h after injury, he developed lethargy; at that time, oxygen saturation was normal, and head CT, chest CT, and CT pulmonary angiography showed no intracranial, pulmonary parenchymal, or pulmonary embolic abnormality. Two hours later, generalized seizures with vomiting were followed by desaturation and intubation, indicating that the subsequent respiratory failure was temporally more consistent with aspiration than primary pulmonary fat embolism. After ICU admission, brain MRI demonstrated the characteristic “starfield” pattern. Day-1 resting right-heart contrast echocardiography showed no definite right-to-left shunt. Management was mainly supportive, including airway protection, mechanical ventilation, continuous EEG monitoring, antiseizure therapy, targeted temperature management, organ support, infection control, and staged orthopedic treatment.

**Outcomes:**

Despite comprehensive treatment, the patient developed refractory status epilepticus, septic shock, rhabdomyolysis, acute kidney injury, hepatic dysfunction, and coagulopathy. Serial early neuron-specific enolase levels remained normal. He remained in an unresponsive wakefulness syndrome at 1 month and had a severe disorder of consciousness with a Glasgow Coma Scale score of 6 at 15 months.

**Conclusion:**

Severe CFES may occur despite normal early pulmonary imaging and oxygenation. Early brain MRI is essential after unexplained neurological deterioration following long-bone fractures. In this case, normal early NSE did not exclude devastating neurological injury.

## 1 Introduction

Fat embolism syndrome (FES) is an uncommon but potentially life-threatening complication most often associated with long-bone fractures, pelvic fractures, and orthopedic procedures. The classic clinical triad consists of respiratory insufficiency or hypoxemia, neurological abnormalities, and petechial rash, usually occurring within 12–72 h after injury (1–3). Because embolized marrow fat first encounters the pulmonary microcirculation, pulmonary manifestations are often regarded as an early and important clinical clue.

Cerebral fat embolism syndrome (CFES) represents the neurological manifestation of FES. Its clinical spectrum ranges from mild confusion and agitation to seizures, coma, diffuse cerebral edema, persistent disorder of consciousness, and death (4–8). Brain magnetic resonance imaging (MRI), especially diffusion-weighted imaging (DWI), is central to diagnosis. The characteristic “starfield” pattern, first described by Parizel et al. (4) consists of multiple punctate DWI hyperintense lesions with restricted diffusion and reflects widespread cerebral microembolization.

Although many patients with CFES improve with supportive care, severe and fatal courses have been reported. Armstrong et al. (6) found that early symptom onset and severe MRI grade were associated with higher mortality in patients with cerebral fat embolism. Rapid-onset CFES progressing to brain death has also been described (8). These observations suggest that CFES is clinically heterogeneous and that early neurological deterioration may identify a severe disease course.

Importantly, CFES does not always follow the classic sequence of pulmonary involvement preceding neurological dysfunction. Cases with isolated or predominant neurological involvement and absent respiratory or dermatological signs have been reported (5, 9, 10). These reports support the clinical plausibility of pulmonary-sparing CFES but do not provide a definitive mechanism for absent early respiratory findings. In pulmonary FES, chest CT commonly shows ground-glass opacities, consolidation, centrilobular nodules, septal thickening, or other parenchymal abnormalities (11–13). Therefore, normal pulmonary imaging and preserved oxygenation may falsely lower clinical suspicion when cerebral manifestations dominate.

The present case is notable for a marked dissociation between cerebral and pulmonary involvement. A young man developed typical CFES after lower-extremity long-bone fractures. At the time of neurological deterioration, oxygenation remained preserved and head CT, chest CT, and CT pulmonary angiography were unremarkable. Respiratory failure occurred only after generalized seizures and vomiting, supporting aspiration rather than primary pulmonary fat embolism as the immediate cause. After ICU admission, brain MRI demonstrated the characteristic DWI “starfield” pattern. Right-heart contrast echocardiography with agitated saline showed no definite right-to-left shunt at rest, although provoked shunting could not be excluded because the Valsalva maneuver was not feasible. Despite comprehensive supportive and adjunctive treatment, the patient developed refractory status epilepticus and persistent severe disorder of consciousness, while serial early neuron-specific enolase (NSE) levels remained normal. This case highlights a pulmonary-sparing, treatment-refractory form of CFES and raises important diagnostic and prognostic considerations.

## 2 Case presentation

### 2.1 Chief complaint

A 23-year-old man presented to the emergency department with severe pain in the left shoulder and right lower extremity after a high-speed traffic accident.

### 2.2 History of present illness and clinical course

#### 2.2.1 . Day 0, approximately 2 h after injury

The patient sustained multiple traumatic injuries, including left shoulder dislocation, right femoral shaft fracture, right open tibiofibular fracture, right medial malleolus fracture, right knee open injury with patellar tendon rupture, and right lower-extremity degloving injury with compartment syndrome. Radiography confirmed fractures of the right femur, tibia, and fibula.

Emergency surgical intervention consisted of debridement of the open tibial fracture, open reduction and external fixation of the tibial fracture, patellar tendon repair, fasciotomy for compartment syndrome, reverse skin grafting of the degloving injury, and closed reduction of the left shoulder dislocation. Postoperatively, he was hemodynamically stable and fully conscious, with a Glasgow Coma Scale (GCS) score of 15. Respiratory function was preserved, and arterial oxygenation was normal on room air.

#### 2.2.2 Day 0, approximately 12 h after injury

**Figure 1:**
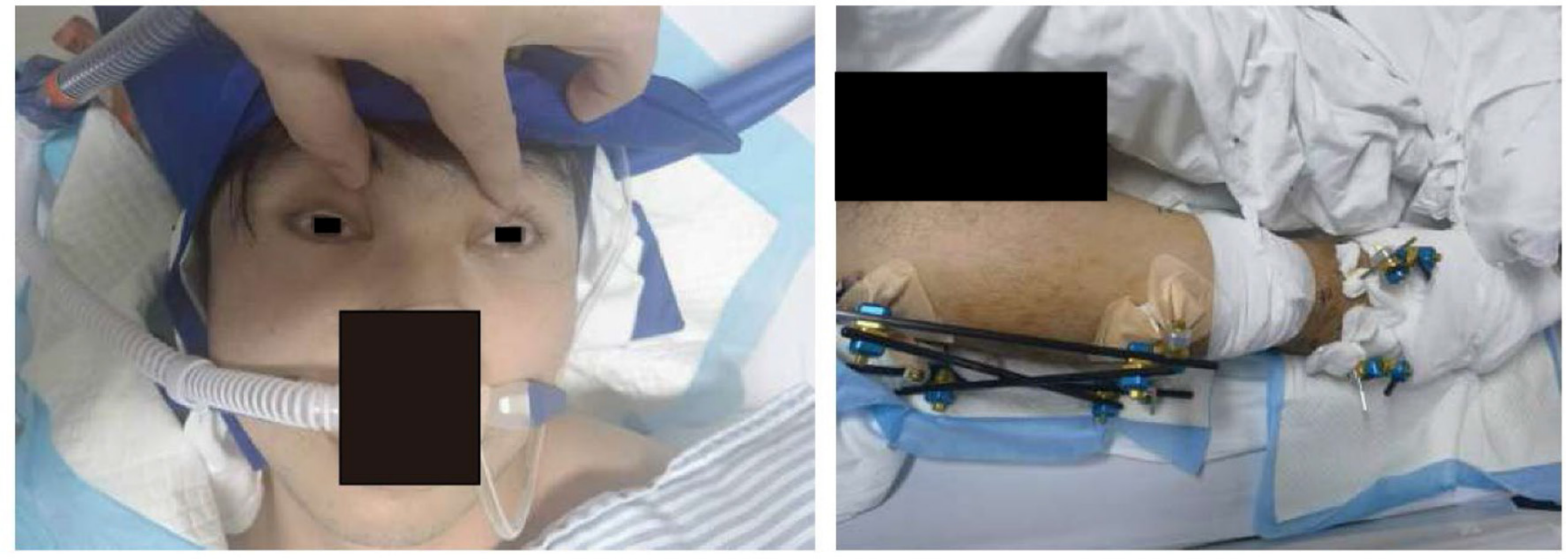
The patient developed lethargy 12 h after injury; however, there was an absence of characteristic petechiae on the body and conjunctiva.

The patient developed acute altered mental status with progressive lethargy. His GCS score decreased to 9 (E2V2M5). At this time, respiratory function remained clinically intact, and oxygen saturation was preserved. No petechial rash was observed on the conjunctiva, oral mucosa, trunk, or extremities (Figure 1).

**Figure 2:**
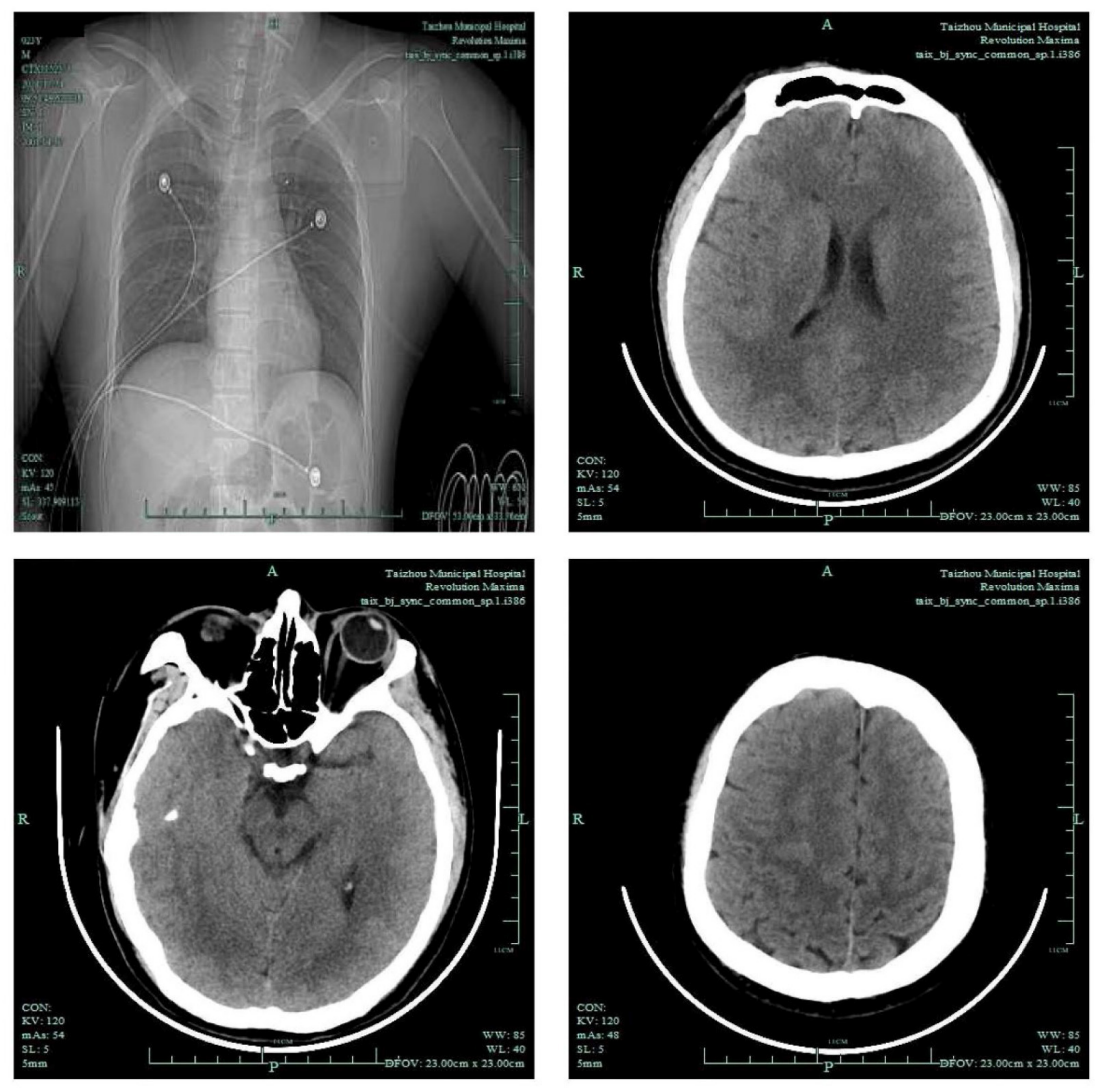
The patient became lethargic 12 h after injury, but follow-up CT scans of the chest and head showed no significant abnormalities.

Head CT showed no acute intracranial hemorrhage, large territorial infarction, or obvious cerebral edema. Chest CT and CT pulmonary angiography showed no evident pulmonary parenchymal lesion, pulmonary embolic abnormality, fat-density intravascular material, consolidation, diffuse ground-glass opacity, or other imaging findings suggestive of primary pulmonary fat embolism (Figure 2). Importantly, this pulmonary assessment was performed at the time of neurological deterioration and before the subsequent seizure-vomiting event.

#### 2.2.3 Day 0, approximately 14 h after injury

Within the following 2 h, the patient developed generalized tonic-clonic seizures accompanied by vomiting and oxygen desaturation to 82% on room air. Emergency endotracheal intubation was performed, and he was transferred to the intensive care unit (ICU). Based on the chronological sequence, the acute respiratory failure was attributed mainly to seizure-associated aspiration rather than primary pulmonary fat embolism.

#### 2.2.4 Day 0, ICU admission, approximately 16 h after injury

**Figure 3:**
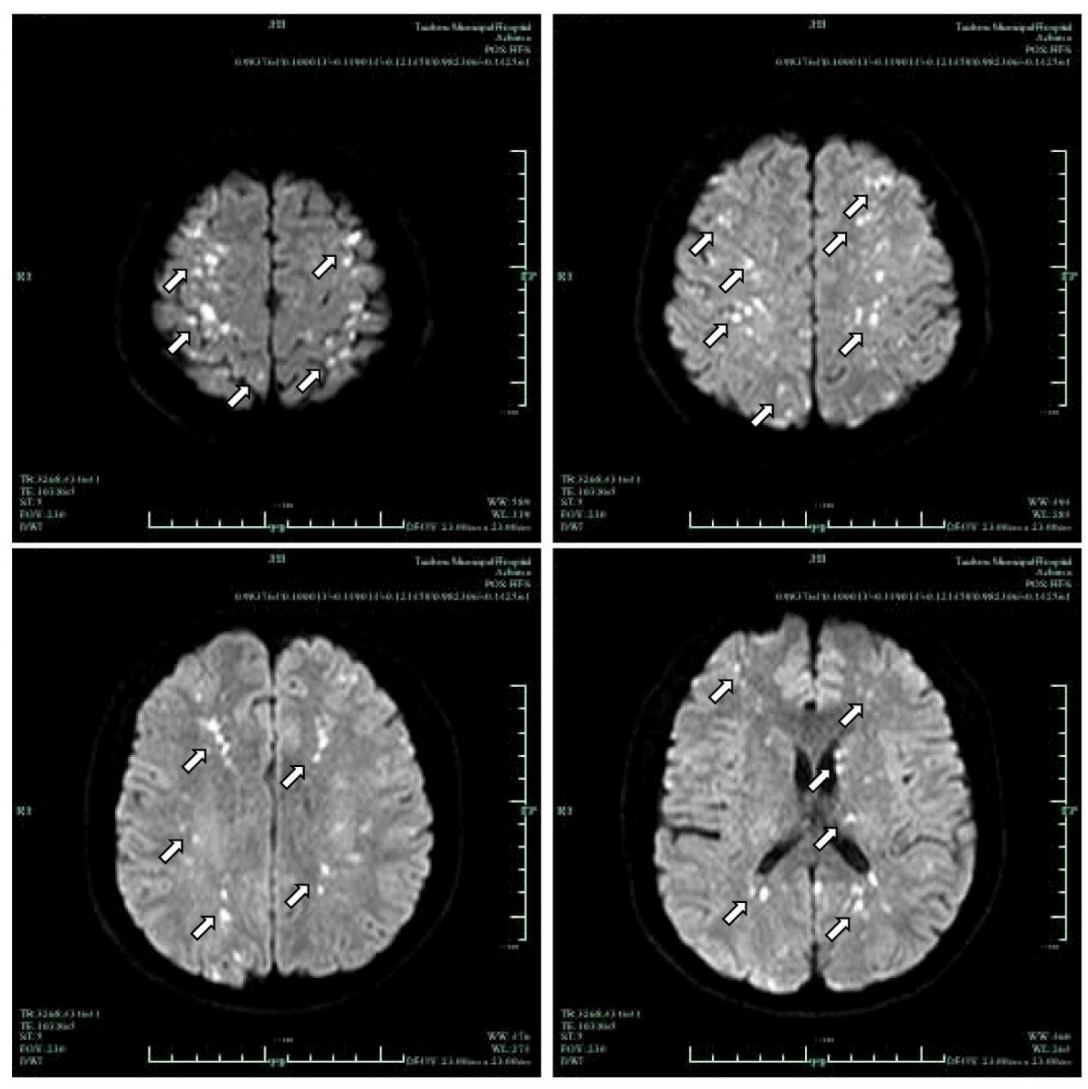
After the patient was transferred to the ICU, a follow-up cranial MRI, including DWI and MRA, revealed the classic “starfield” pattern. The MRA showed no evidence of large-vessel occlusion, leading to a diagnosis of cerebral fat embolism.

Brain MRI with DWI and magnetic resonance angiography (MRA) demonstrated multiple scattered punctate hyperintense lesions with restricted diffusion throughout both cerebral hemispheres, basal ganglia, thalami, and cerebellum. These findings were consistent with the characteristic “starfield” pattern of CFES. MRA showed no evidence of large-vessel occlusion. Susceptibility-weighted imaging showed no hemorrhagic transformation (Figure 3).

#### 2.2.5 Day 1, right-heart contrast echocardiography

Right-heart contrast echocardiography with agitated saline was performed to screen for right-to-left shunt. The patient was deeply unconscious and mechanically ventilated and therefore could not perform the Valsalva maneuver. Only resting-state assessment was completed. No definite passage of microbubbles into the left cardiac chambers was observed at rest. This finding did not support a significant resting right-to-left shunt. However, a small or provoked patent foramen ovale-related shunt could not be completely excluded because provocative maneuvers were not feasible. Transesophageal echocardiography was not performed during the acute phase. Therefore, the negative resting study reduced the likelihood of a large resting shunt but could not eliminate transient or provoked right-to-left passage.

#### 2.2.6 ICU course, Days 1–11

The patient developed refractory status epilepticus. The patient met criteria for refractory status epilepticus, with persistent electrographic seizures despite continuous benzodiazepine-based sedation and appropriately dosed intravenous antiseizure medications. Continuous electroencephalographic monitoring showed periodic epileptiform discharges. Targeted temperature management at 34°C was initiated after ICU admission and maintained for 48 h, but no meaningful neurological improvement was observed. Intravenous dexamethasone 40 mg daily was started on Day 1, tapered every 3 days, and discontinued after 9 days.

The subsequent ICU course was complicated by septic shock requiring norepinephrine at a maximum dose of 0.8 μg/kg/min and vasopressin, severe rhabdomyolysis with a peak creatine kinase level of 12,450 U/L, acute kidney injury classified as KDIGO stage 3 requiring continuous renal replacement therapy from Day 4 to Day 12, hepatic dysfunction with predominant indirect hyperbilirubinemia, and coagulopathy with markedly elevated D-dimer and mild thrombocytopenia.

#### 2.2.7 Day 8

Follow-up non-contrast head CT showed evolving hypodensity in the bilateral centrum semiovale and subcortical white matter, consistent with established ischemic injury and resolving cerebral edema. Given the persistent severe impairment of consciousness and the anticipated need for prolonged airway support, tracheostomy was performed on the same day.

#### 2.2.8 Day 12

After hemodynamic stabilization and partial correction of coagulopathy, the patient underwent further orthopedic surgery, including removal of the tibial external fixator, wound debridement, open reduction and internal fixation of the femoral shaft fracture with bone grafting, tendon repair, lower-leg wound debridement, placement of an antibiotic-impregnated bone cement spacer, and chronic ulcer wound repair.

#### 2.2.9 Day 17

Additional wound debridement, surgical drainage, and chronic ulcer wound repair were performed.

#### 2.2.10 . Outcome

At approximately 1 month after injury, the patient remained in a unresponsive wakefulness syndrome, with eye opening to pain, no verbal response because of tracheostomy, and decerebrate posturing. The GCS score was 4 (E2V1M1). He was transferred to a local hospital for continued rehabilitation and long-term care.

Telephone follow-up at 15 months after injury indicated persistent severe disorder of consciousness, with a reported GCS score of 6 (E2V1M3).

**Figure 4:**
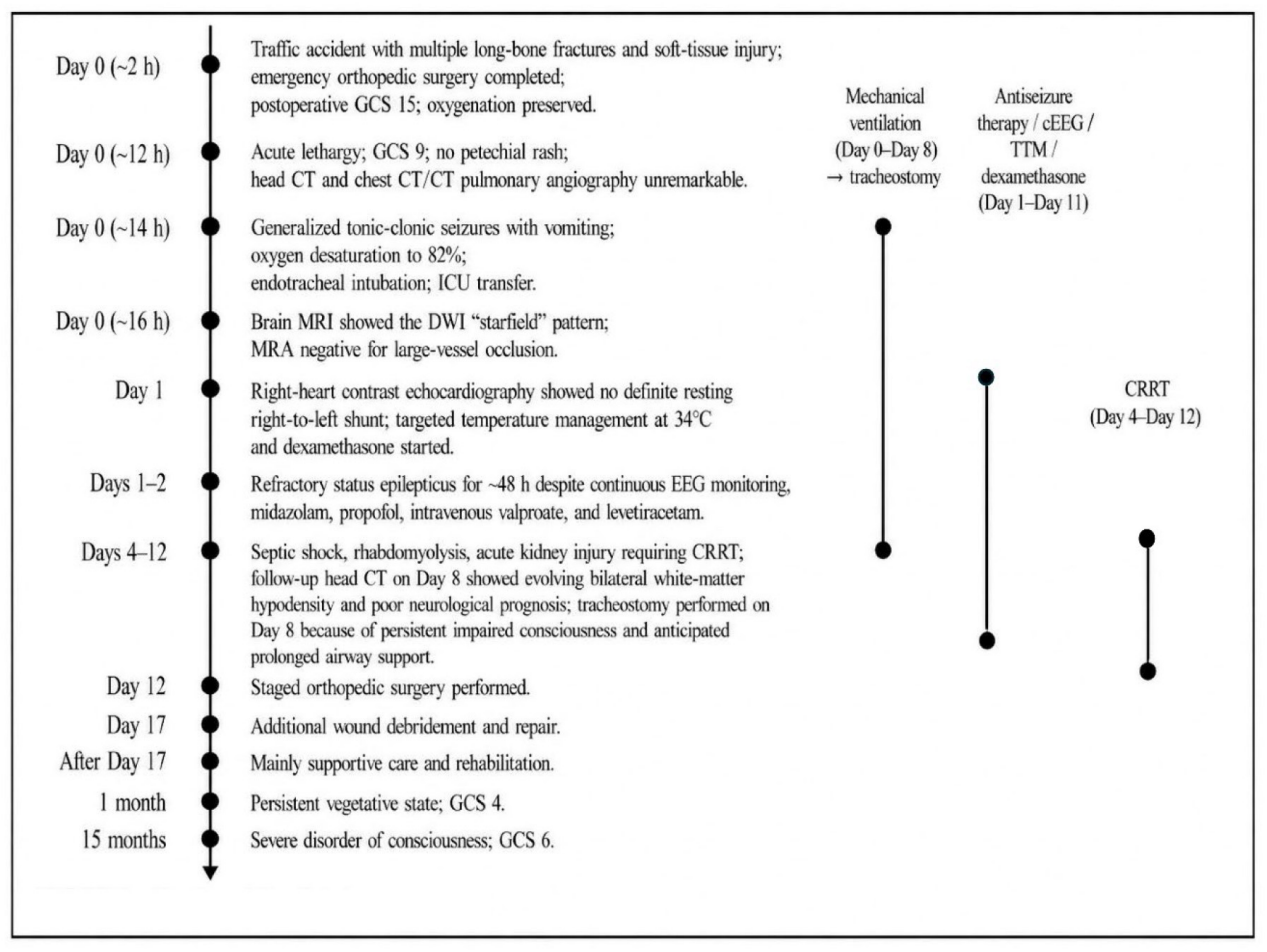
Timeline of the patient's clinical course.

The overall clinical course is summarized in Figure 4.

### 2.3 Past medical history

The patient had no significant prior medical history, regular medication use, or known drug allergies.

### 2.4 Physical examination on ICU admission

On ICU admission, the patient was intubated and sedated. His temperature was 38.2°C, heart rate was 118 beats/min, and blood pressure was 128/62 mmHg with norepinephrine at 0.15 μg/kg/min. Pulmonary auscultation revealed coarse breath sounds bilaterally without obvious rales or wheezes. Oxygen saturation was 100% under an FiO_2_ of 30%. Neurological examination showed a left-sided Babinski sign and increased muscle tone in all extremities. No petechial hemorrhages were observed on the conjunctiva, oral mucosa, trunk, or extremities.

This oxygenation profile was clinically important. Although intubation had been required after seizures and vomiting, the patient maintained normal oxygen saturation and PaO_2_ with only 30% inspired oxygen shortly after ICU admission. Together with the absence of pulmonary CT and CT pulmonary angiography abnormalities at the time of neurological deterioration, this supported aspiration-related respiratory compromise rather than primary pulmonary fat embolism as the immediate cause of respiratory failure.

### 2.5 Laboratory studies

A comprehensive laboratory evaluation was performed on ICU admission, approximately 16 h after injury. Key laboratory findings on ICU admission are summarized in Table 1.

**Table 1:** Key laboratory findings on ICU admission.

Parameter	Value	Reference range	Unit
Blood gas analysis (FiO_2_ 0.30)			
pH	7.445	7.35–7.45	
PaO_2_	102.0	80–100	mmHg
PaCO_2_	32.4	35–45	mmHg
Lactate	2.1	0.5–1.6	mmol/L
${\text{HCO}}_{3}^{-}$	21.9	22–26	mmol/L
Base excess	−2.3	−2.0 to +2.0	mmol/L
PaO_2_/FiO_2_ ratio	340	>300	mmHg
Hematology			
White blood cell count	16.9	4.0–10.0	×10^9^/L
Neutrophils	93.7	50–70	%
Lymphocytes	4.1	20–40	%
Monocytes	2.0	3–8	%
Hemoglobin	105.0	130–175	g/L
Platelet count	196.0	125–350	×10^9^/L
Biochemistry			
Glucose	7.38	3.9–6.1	mmol/L
Total bilirubin	42.4	3.4–20.5	μmol/L
Direct bilirubin	9.3	0–6.8	μmol/L
Indirect bilirubin	33.1	1.7–13.7	μmol/L
Albumin	37.9	40–55	g/L
Creatine kinase	3,223.0	38–174	U/L
Lactate dehydrogenase	549.0	120–250	U/L
α-Hydroxybutyrate dehydrogenase	316.0	72–182	U/L
Blood urea nitrogen	7.7	2.6–7.5	mmol/L
Creatinine	85.9	57–97	μmol/L
Potassium	4.0	3.5–5.3	mmol/L
Sodium	138.0	136–145	mmol/L
Chloride	106.7	98–106	mmol/L
Calcium	1.95	2.10–2.55	mmol/L
Magnesium	0.73	0.75–1.25	mmol/L
Phosphorus	0.90	0.87–1.45	mmol/L
Coagulation			
Prothrombin time	12.6	11.0–14.0	s
Activated partial thromboplastin time	26.6	25.0–35.0	s
Fibrinogen	3.30	2.00–4.00	g/L
D-dimer	4.57	< 0.50	mg/L
Cardiac biomarkers			
Myoglobin	1,129.0	25–72	μg/L
Creatine kinase-MB	6.6	< 25	ng/mL
Troponin T	0.015	< 0.014	μg/L
B-type natriuretic peptide	77.7	< 100	ng/L
Inflammatory markers			
C-reactive protein	68.82	< 10	mg/L
Procalcitonin	0.13	< 0.05	ng/mL
Neuronal injury marker			
Neuron-specific enolase	15.9	< 16.3	ng/mL

Arterial blood gas analysis under FiO_2_ 0.30 showed a pH of 7.445, PaCO_2_ of 32.4 mmHg, PaO_2_ of 102.0 mmHg, lactate of 2.1 mmol/L, ${\text{HCO}}_{3}^{-}$ of 21.9 mmol/L, base excess of −2.3 mmol/L, and PaO_2_/FiO_2_ ratio of 340 mmHg. Hematologic testing revealed leukocytosis with neutrophilia, mild anemia, and a normal platelet count. Serum biochemistry showed elevated creatine kinase, lactate dehydrogenase, α-hydroxybutyrate dehydrogenase, and indirect bilirubin. Coagulation testing demonstrated elevated D-dimer with normal fibrinogen at ICU admission.

Serial early NSE levels remained within the normal range despite subsequent poor neurological outcome: 15.9 ng/mL on Day 1, 8.6 ng/mL on Day 3, 6.3 ng/mL on Day 5, and 8.9 ng/mL on Day 7.

### 2.6 Final diagnoses

Pulmonary-sparing fulminant cerebral fat embolism syndrome; refractory status epilepticus; aspiration-related respiratory failure; multiple lower-extremity long-bone fractures with extensive soft-tissue injury; severe rhabdomyolysis; septic shock; acute kidney injury; hepatic dysfunction.

## 3 Discussion

The present case highlights a rare pulmonary-sparing form of fulminant CFES after long-bone fractures. Classic FES is commonly recognized by the triad of hypoxemia, neurological abnormalities, and petechial rash (1–3). In contrast, this patient developed abrupt neurological deterioration while oxygenation, chest CT, and CT pulmonary angiography remained unremarkable, and no petechial rash was observed. Respiratory failure occurred only after generalized seizures and vomiting, and oxygenation was rapidly maintained after intubation with an FiO_2_ of 30%. Therefore, the early clinical course was more consistent with predominant cerebral involvement and secondary aspiration-related respiratory compromise than with classic pulmonary FES followed by hypoxic neurological injury. The main issues raised by this case are why severe cerebral involvement occurred without early pulmonary manifestations, how CFES was distinguished from diffuse axonal injury, and why normal early NSE levels did not predict a favorable neurological outcome.

### 3.1 Why were pulmonary imaging and oxygenation initially normal?

Our interpretation is cautious: the early normal oxygenation and thoracic imaging do not prove that fat never reached the pulmonary microcirculation; rather, they indicate the absence of clinically detectable pulmonary FES at the time of neurological deterioration. In the mechanical model, marrow fat released after fracture or orthopedic manipulation enters the venous circulation. Larger fat droplets are usually trapped in the pulmonary capillary bed, whereas smaller or more deformable droplets may pass through the pulmonary microcirculation or enter the systemic circulation through an intracardiac right-to-left shunt (14–16). Cerebral fat embolism has also been reported in the absence of an intracardiac shunt, supporting the possibility of transpulmonary passage or non-shunt mechanisms in selected patients (16).

Normal chest CT and CT pulmonary angiography do not exclude microscopic or transient pulmonary fat embolization. This is particularly relevant because fat embolism may involve the pulmonary microcirculation and may not always produce macroscopic parenchymal abnormalities detectable on early thoracic imaging (14, 17). Pulmonary FES may present with ground-glass opacities, consolidation, centrilobular nodules, septal thickening, or other parenchymal abnormalities when lung injury is clinically evident (11–13). In this patient, however, pulmonary imaging and oxygenation were normal at the time of neurological deterioration. The later hypoxemia occurred only after generalized seizures and vomiting, and oxygenation was rapidly maintained with low inspired oxygen after intubation, supporting aspiration rather than primary pulmonary fat embolism as the immediate cause of respiratory failure.

Biochemical injury may further explain this brain-lung dissociation. Hydrolysis of neutral fat into free fatty acids can induce endothelial injury, increased capillary permeability, inflammatory activation, and microthrombosis (14, 15). These processes may amplify cerebral microvascular injury even when pulmonary manifestations are clinically silent. The brain is highly vulnerable to diffuse microvascular obstruction and endothelial dysfunction, which may explain the extensive DWI “starfield” pattern despite initially normal thoracic imaging.

Right-heart contrast echocardiography showed no definite right-to-left shunt at rest. However, the patient could not perform the Valsalva maneuver because of impaired consciousness and mechanical ventilation. Since provocative maneuvers can improve the detection of intermittent right-to-left shunts during agitated saline contrast echocardiography, a small or provoked shunt could not be completely excluded (18, 19). Nevertheless, CFES can occur without a demonstrable intracardiac shunt, and cerebral fat embolism without intracardiac shunting has been reported (16).

Previously reported pulmonary-sparing or pulmonary-silent CFES cases support this clinical pattern, including reports without respiratory or dermatological involvement and reports without demonstrable intracardiac shunting (5, 9, 10, 16). Compared with those reports, the educational value of our case lies in the documented sequence: normal oxygenation and thoracic imaging at the time of mental status change, followed later by seizure-vomiting-associated respiratory failure.

### 3.2 Diagnostic reasoning and distinction from diffuse axonal injury

A major diagnostic challenge in this case was distinguishing CFES from diffuse axonal injury (DAI), another important cause of impaired consciousness after high-energy trauma. DAI results from traumatic acceleration-deceleration and rotational shearing forces and is one of the important pathological substrates of impaired consciousness after traumatic brain injury (20, 21). Typical lesions are often found in the gray-white matter junction, corpus callosum, and brainstem, and susceptibility-sensitive sequences are particularly useful for detecting hemorrhagic microlesions (21, 22). Moreover, DAI can be under-recognized on early CT, and MRI is generally more informative for detecting traumatic axonal lesions, although diagnostic limitations remain (23).

Several features favored CFES rather than DAI in this patient. First, the patient was fully conscious after emergency surgery, with a GCS score of 15, and then developed delayed neurological deterioration approximately 12 h after injury. This temporal pattern is more compatible with CFES than with severe DAI, which usually causes immediate post-traumatic neurological impairment. Second, brain MRI showed multiple scattered punctate DWI hyperintense lesions with restricted diffusion involving both cerebral hemispheres, basal ganglia, thalami, and cerebellum, forming the characteristic “starfield” pattern of CFES (4, 7). Third, MRA showed no large-vessel occlusion, and susceptibility-weighted imaging showed no hemorrhagic transformation, making major vascular occlusion and hemorrhagic shear injury less likely. Finally, neurological deterioration preceded aspiration-related hypoxemia, arguing against hypoxic-ischemic injury as the initial mechanism.

Thus, the diagnosis was supported by the convergence of high-risk long-bone trauma, delayed but early neurological decline, initially preserved pulmonary status, and the typical MRI pattern. In similar cases, early brain MRI should be pursued when unexplained neurological deterioration occurs after long-bone fractures, even if head CT and pulmonary evaluation are initially unrevealing.

### 3.3 Normal early NSE despite poor neurological outcome

Another important feature of this case was the discrepancy between normal early neuron-specific enolase (NSE) levels and poor neurological outcome. NSE has been studied as a prognostic biomarker in post-cardiac arrest brain injury, traumatic brain injury, and ischemic stroke (24–26). However, its diagnostic or prognostic value in CFES has not been established. Therefore, extrapolating NSE thresholds from cardiac arrest, traumatic brain injury, or ischemic stroke to CFES may be inappropriate.

In this patient, serial early NSE levels remained within the normal range despite extensive DWI abnormalities, refractory status epilepticus, unresponsive wakefulness syndrome at 1 month, and severe disorder of consciousness at 15 months. This discrepancy may reflect the pathobiology of CFES. Unlike a large territorial infarction or global hypoxic-ischemic injury, CFES often produces multifocal punctate microvascular lesions, endothelial dysfunction, blood-brain barrier disruption, cytotoxic edema, and inflammatory neurovascular injury. Early neuronal cytoplasmic release may therefore be insufficient, delayed, or not well reflected by serum NSE levels.

This observation should be interpreted cautiously. It does not prove that NSE has no value in CFES, but it suggests that normal early NSE should not be considered reassuring when the clinical course and MRI pattern indicate severe cerebral injury. At present, neurological examination, MRI lesion burden, EEG evolution, and long-term clinical trajectory remain more informative than NSE alone. Further cases are needed to clarify whether NSE has reproducible prognostic value in CFES.

### 3.4 Study limitations

This report has several limitations. First, it describes a single patient, and the findings should be generalized cautiously. Second, pathological confirmation of fat emboli in the brain or lung was not available. Third, right-heart contrast echocardiography was limited to the resting state because the Valsalva maneuver could not be performed, and transesophageal echocardiography was not performed during the acute phase. Fourth, normal early chest CT and CT pulmonary angiography cannot exclude microscopic pulmonary fat embolization. Fifth, although NSE is used in other forms of acute brain injury, its role in CFES remains uncertain; the normal early NSE values in this case should therefore be considered hypothesis-generating rather than definitive evidence.

## 4 Conclusion

This case highlights an initially pulmonary-sparing form of fulminant CFES after long-bone fractures. Severe cerebral involvement may occur despite normal early pulmonary imaging and preserved oxygenation, and the characteristic DWI “starfield” pattern remains critical for diagnosis when neurological deterioration is otherwise unexplained. In this case, normal early NSE did not reflect the severity of neurological injury and should therefore be interpreted cautiously.

## Data Availability

The original contributions presented in the study are included in the article/supplementary material, further inquiries can be directed to the corresponding author.

## References

[1] Gurd AR Wilson RI. The fat embolism syndrome. J Bone Joint Surg Br. (1974) 56b:408–16. doi: 10.1302/0301-620X.56B3.408 4547466

[2] Mellor A Soni N. Fat embolism. Anaesthesia. (2001) 56:145–54. doi: 10.1046/j.1365-2044.2001.01724.x 11167474

[3] Shaikh N. Emergency management of fat embolism syndrome. J Emerg Trauma Shock. (2009) 2:29–33. doi: 10.4103/0974-2700.44680 19561953 PMC2700578

[4] Parizel PM Demey HE Veeckmans G Verstreken F Cras P Jorens PG . Early diagnosis of cerebral fat embolism syndrome by diffusion-weighted MRI (starfield pattern). Stroke. (2001) 32:2942–4. doi: 10.1161/str.32.12.2942 11740000

[5] Vetrugno L Bignami E Deana C Bassi F Vargas M Orsaria M . Cerebral fat embolism after traumatic bone fractures: a structured literature review and analysis of published case reports. Scand J Trauma Resusc Emerg Med. (2021) 29:47. doi: 10.1186/s13049-021-00861-x 33712051 PMC7953582

[6] Armstrong BRW Devendra A Pokale S Subramani B Rajesh Babu V Ramesh P . Can the rate of mortality and neurological recovery be predicted from the time of onset of symptoms and MRI grade in patients with cerebral fat embolism?: A study of 34 patients. Bone Joint J. (2022) 104-b:142–9. doi: 10.1302/0301-620X.104B1.BJJ-2021-0420.R1 34969291

[7] Kuo KH Pan YJ Lai YJ Cheung WK Chang FC Jarosz J. Dynamic MR imaging patterns of cerebral fat embolism: a systematic review with illustrative cases. Am J Neuroradiol. (2014) 35:1052–7. doi: 10.3174/ajnr.A3605 23639561 PMC7965147

[8] Williams JD Gates JD Croteau AJ. Rapid-onset cerebral fat embolism syndrome leading to brain death: a case report. Trauma Case Rep. (2024) 54:101109. doi: 10.1016/j.tcr.2024.101109 39411451 PMC11474175

[9] Salimi Z Ami Ali M Tazi R Mimouni Y Hazim A Aasfara J . Rare case of cerebral fat embolism with no respiratory or dermatologic involvement. Cureus. (2022) 14:e22192. doi: 10.7759/cureus.22192 35308671 PMC8926028

[10] Algahtani HA Shirah BH Abdelghaffar N Alahmari F Alhadi W Alqahtani SA. Cerebral fat embolism syndrome: diagnostic challenges and catastrophic outcomes: a case series. J Yeungnam Med Sci. (2023) 40:207–11. doi: 10.12701/jyms.2022.00360 36069083 PMC10076914

[11] Piolanti M Dalpiaz G Scaglione M Coniglio C Miceli M Violini S . Fat embolism syndrome: lung computed tomography findings in 18 patients. J Comput Assist Tomogr. (2016) 40:335–42. doi: 10.1097/RCT.0000000000000376 26938691

[12] Qi M Zhou H Yi Q Wang M Tang Y. Pulmonary CT imaging findings in fat embolism syndrome: case series and literature review. Clin Med. (2023) 23:88–93. doi: 10.7861/clinmed.2022-0428 36697017 PMC11046538

[13] Nucifora G Hysko F Vit A Vasciaveo A. Pulmonary fat embolism: common and unusual computed tomography findings. J Comput Assist Tomogr. (2007) 31:806–7. doi: 10.1097/rct.0b013e318032566e 17895796

[14] Hulman G. The pathogenesis of fat embolism. J Pathol. (1995) 176:3–9. doi: 10.1002/path.1711760103 7616354

[15] Taviloglu K Yanar H. Fat embolism syndrome. Surg Today. (2007) 37:5–8. doi: 10.1007/s00595-006-3307-5 17186337

[16] Eriksson EA Schultz SE Cohle SD Post KW. Cerebral fat embolism without intracardiac shunt: a novel presentation. J Emerg Trauma Shock. (2011) 4:309–12. doi: 10.4103/0974-2700.82233 21769222 PMC3132375

[17] Jorens PG Van Marck E Snoeckx A Parizel PM. Nonthrombotic pulmonary embolism. Eur Respir J. (2009) 34:452–74. doi: 10.1183/09031936.00141708 19648522

[18] Pinto FJ. When and how to diagnose patent foramen ovale. Heart. (2005) 91:438–40. doi: 10.1136/hrt.2004.052233 15772190 PMC1768819

[19] Marriott K Manins V Forshaw A Wright J Pascoe R. Detection of right-to-left atrial communication using agitated saline contrast imaging: experience with 1162 patients and recommendations for echocardiography. J Am Soc Echocardiogr. (2013) 26:96–102. doi: 10.1016/j.echo.2012.09.007 23072711

[20] Fernández-Torre JL Burgueño P Ballesteros MA Hernández-Hernández MA Villagrá-Terán N de Lucas EM. Super-refractory nonconvulsive status epilepticus secondary to fat embolism: a clinical, electrophysiological, and pathological study. Epilepsy Behav. (2015) 49:184–8. doi: 10.1016/j.yebeh.2015.04.045 25986321

[21] Smith DH Meaney DF Shull WH. Diffuse axonal injury in head trauma. J Head Trauma Rehabil. (2003) 18:307–16. doi: 10.1097/00001199-200307000-00003 16222127

[22] Tao JJ Zhang WJ Wang D Jiang CJ Wang H Li W . Susceptibility weighted imaging in the evaluation of hemorrhagic diffuse axonal injury. Neural Regen Res. (2015) 10:1879–81. doi: 10.4103/1673-5374.170322 26807130 PMC4705807

[23] Jang SH. Diagnostic problems in diffuse axonal injury. Diagnostics. (2020) 10:117. doi: 10.3390/diagnostics10020117 32098060 PMC7168326

[24] Stammet P Collignon O Hassager C Wise MP Hovdenes J Åneman A . Neuron-specific enolase as a predictor of death or poor neurological outcome after out-of-hospital cardiac arrest and targeted temperature management at 33 °C and 36 °C. J Am Coll Cardiol. (2015) 65:2104–14. doi: 10.1016/j.jacc.2015.03.538 25975474

[25] Cheng F Yuan Q Yang J Wang W Liu H. The prognostic value of serum neuron-specific enolase in traumatic brain injury: systematic review and meta-analysis. PLoS ONE. (2014) 9:e106680. doi: 10.1371/journal.pone.0106680 25188406 PMC4154726

[26] Mochetti MM Silva EGP Correa AAF Cabette MR Perissinotti IN Loj ES . Neuron-specific enolase at admission as a predictor for stroke volume, severity and outcome in ischemic stroke patients: a prognostic biomarker review. Sci Rep. (2024) 14:2688. doi: 10.1038/s41598-024-53080-6 38302568 PMC10834412

